# New intraocular pressure measurement method using reflected pneumatic pressure from cornea deformed by air puff of ring-type nozzle

**DOI:** 10.1371/journal.pone.0186738

**Published:** 2017-12-07

**Authors:** Hyung Jin Kim, Yeong Ho Seo, Byeong Hee Kim

**Affiliations:** Department of Mechanical and Mechatronics Engineering, Kangwon National University, Chuncheon, Republic of Korea; Bascom Palmer Eye Institute, UNITED STATES

## Abstract

In this study, a non-contact type intraocular pressure (IOP) measuring system using reflected pneumatic pressure is proposed to overcome the disadvantages of existing measurement systems. A ring-type nozzle, a key component in the proposed system, is designed via computational fluid analysis. It predicts the reflected pneumatic pressure based on the nozzle exit angle and inner and outer diameters of the nozzle, which are 30°, 7 mm, and 9 mm, respectively. Performance evaluation is conducted using artificial eyes fabricated using polydimethylsiloxane with the specifications of human eyes. The IOP of the fabricated artificial eyes is adjusted to 10, 30, and 50 mm Hg, and the reflected pneumatic pressure is measured as a function of the distance between the ring-type nozzle and artificial eye. The measured reflected pneumatic pressure is high when the measurement distance is short and eye pressure is low. The cornea of an artificial eye is significantly deformed at a low IOP, and the applied pneumatic pressure is more concentrated in front of the ring-type nozzle because of the deformed cornea. Thus, the reflected pneumatic pressure at a low IOP has more inflows into the pressure sensor inserted inside the nozzle. The sensitivity of the output based on the IOP at measurement distances between 3–5 mm is -0.0027, -0.0022, -0.0018, -0.0015, and -0.0012. Sensitivity decreases as the measurement distance increases. In addition, the reflected pneumatic pressure owing to the misalignment at the measurement distances of 3–5 mm is not affected within a range of 0.5 mm. Therefore, the measurement range is acceptable up to a 1 mm diameter from the center of an artificial eye. However, the accuracy gradually decreases as the reflected pneumatic pressure from a misalignment of 1 mm or more decreases by 26% or more.

## Introduction

Intraocular pressure (IOP) is the pressure inside the eyes needed to maintain their normal structure and function [1]. The average IOP of humans is approximately 15 mm Hg, and a pressure of 10–21 mm Hg is considered as the IOP of normal range [2–5]. Increase in IOP is a major cause of glaucoma. Such an increase occurs either when the aqueous humor of an eye is overproduced inside the anterior chamber between the cornea and iris or when the discharge is not smooth. When the pressure inside an eye rises, the optic nerve is pressed or the blood supply becomes disturbed, and glaucoma is caused by the resulting abnormal optic nerve function [6]. Once the optic nerve is damaged, recovery is almost impossible, and if it is not properly treated before the damage sets in, then the sight is lost [7].

**Fig 1:**
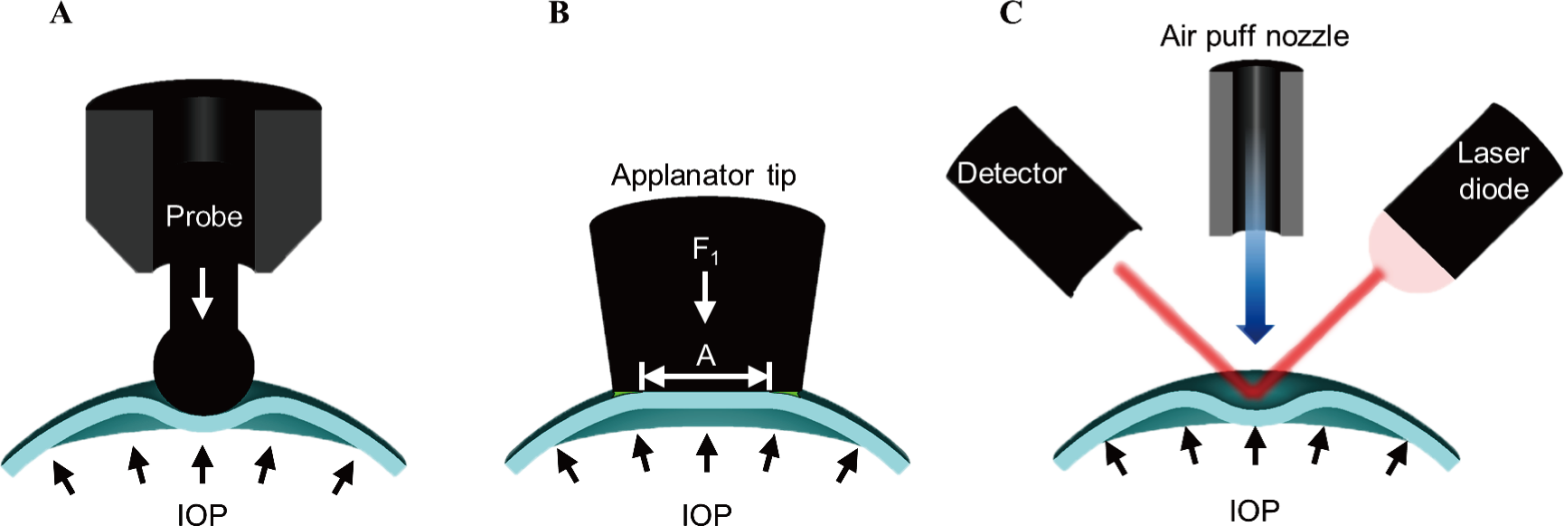
Working principle of conventional intraocular pressure measuring system. (a) Rebound tonometer. (b) Applanation tonometer. (c) Air-puff tonometer.

The most commonly used method for diagnosing glaucoma is measurement of the IOP [8]. It can be seen from Fig 1 that the techniques for measuring the IOP can be classified as probe-rebound, applanation, and non-contact type. Fig 1A depicts an IOP measurement method of the probe-rebound type developed by Dekking and Coster in 1967 [9]. This method is based on the principle of rebound measurement in which the cornea is momentarily contacted with a very light circular plastic probe [10]. The probe-rebound type method measures operating parameters such as probe speed and corneal contact time via an induction-based coil system. If the IOP is high, the probe splashes faster and contact time with the cornea is shortened [11]. The IOP is calculated using operating parameters of the probe. This method does not require eye anesthesia and can be measured by a non-expert [12]. However, the implementation of the method is expensive, and the probe may also fall if the equipment is facing downward. Therefore, it can be used only for patients in an upright position.

The applanation type IOP measurement method shown in Fig 1B was developed by Goldman, a Swiss ophthalmologist in 1955 to compensate for the disadvantages of an indentation type system [13]. This method in which a pressure body is brought into direct contact with an eyeball determines the IOP as the force F_1_ required to flatten a constant area (3.06 mm^2^) of the cornea divided by the flattened area [14]. It is now known to be the most accurate and precise method and is being used as the gold standard for IOP measurement [15]. However, this method requires high proficiency and eye anesthesia, and the error caused by a high concentration of fluorescein and possible infection of the contact surface of an eyeball may be some problems associated with it [12, 16].

A non-contact IOP measurement method was first developed by Grolman in 1972 [17]. As displayed in Fig 1C, this method consists of a pneumatic module for corneal deformation and an optical module to measure its. The laser diode and detector of the optical module face each other and are placed obliquely at an angle to the circular eye. When light is irradiated using a laser diode, the reflected light spreads on the surface of the circular eye, and the intensity of light measured by a photodetector is low [18]. However, when the pneumatic pressure applied in the center of the cornea is slowly increased, the cornea becomes more flattened, and the amount of light entering the detector is maximized when the eye is maximally flat. The shape of the cornea is restored to its original shape over time by an elastic restoration force, and the amount of light entering the detector is reduced again [19–21]. Output of the change in the amount of light with time has a pulse shape, and output values measured after μs intervals are converted into mmHg units [22]. This method has a short measurement time, is simple, and does not require anesthesia, but it is very expensive and difficult to miniaturize [23].

A periodic eye test is essential because the sight loss caused by glaucoma can be prevented if its symptoms are detected and treated early [24]. Therefore, the development of a portable self-diagnostic medical system that can proactively diagnose glaucoma at home will enable periodic eye examinations and early detection of glaucoma, and minimize the disease damage. In this study, we have proposed a new portable non-contact IOP measurement method that can overcome the shortcomings of conventional IOP measuring systems and can evaluate the prognosis of glaucoma. A ring-type nozzle was used because the proposed measurement method involves measuring the reflected pneumatic pressure. The ring-type nozzle was designed and fabricated via computational fluid dynamics (CFD) analysis, and was verified through experiments. Since the experiment using eye of the actual human in the verification step of the newly proposed method is contrary to the research ethics, experiments were performed using artificial eyes with elastic modulus and dimension similar to human eyes.

## Materials and methods

### Principle of intraocular pressure measurement

The IOP measurement system proposed in this study uses the reflective pneumatic distance measurement principle of a pneumatic micrometer. This measurement system has a structure in which the ring-type nozzle is arranged concentrically with a two nozzle of a different size. When the pneumatic pressure injected constantly from the external nozzle is applied onto an object, a part of the pneumatic pressure flows out of the object, while the remaining part is reflected by the object and flows into the internal nozzle. At this point, the latter is measured by a pressure sensor inserted into the nozzle, and then converted into a distance.

**Fig 2:**
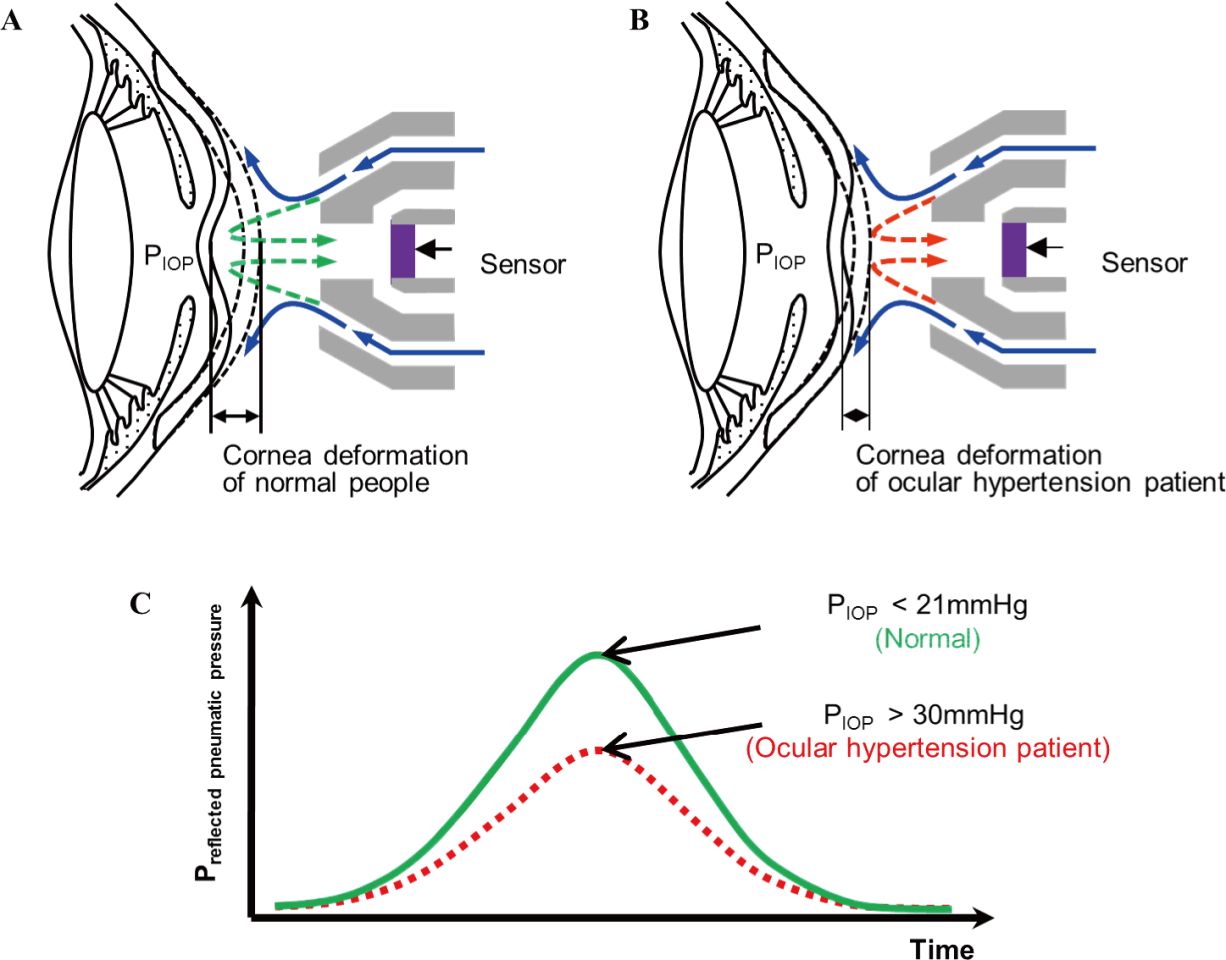
Working principle of the intraocular pressure (IOP) measurement using the proposed system. (a) Corneal deformation (large deformation) of normal person. (b) Corneal deformation (small deformation) of ocular hypertension patient. (c) Comparison of air-rebounding pressure according to corneal deformation.

Fig 2 shows the principle of operation of the proposed IOP measurement system. Fig 2A and 2B illustrate the flow of the reflected pneumatic pressure due to the corneal deformation when a pneumatic pressure is applied into the eyes of a normal person and ocular hypertension patient using the proposed ring-type nozzle [25]. When the pneumatic pressure is injected through the ring-type nozzle, the IOP of a normal person is lower than that of the ocular hypertension patient, so that the corneal deformation for the normal person is larger than that for the ocular hypertension patient. Therefore, ocular hypertension can be diagnosed using the magnitude of the reflected pneumatic pressure as a function of the amount of deformation of the cornea. Fig 2C displays a plot that predicts the amount of reflected pneumatic pressure corresponding to the degree of corneal deformation in a normal person and in the ocular hypertension patient using a pressure sensor inserted in the ring-type nozzle.

### Design of nozzle exit angle

An analysis program used to design the ring-type nozzle to deform the cornea and measure the corneal deformation with the commercial software CFD-ACE+ (ESI Group). In this study, when the pneumatic pressure to deform the cornea was injected through the ring-type nozzle, CFD analysis was performed corresponding to the change in the exit angle (θ) of the ring-type nozzle for an effective reflected pneumatic pressure measurement. In order to investigate the relationship between the area over which the pneumatic pressure is applied with the nozzle and reflected pneumatic pressure, a CFD analysis was conducted based on the gap between the internal and external diameters of the nozzle. The ring-type nozzle used in the analysis was designed as a three-dimensional off lattice structure with 33,311 nodes and 147,924 cells.

**Fig 3:**
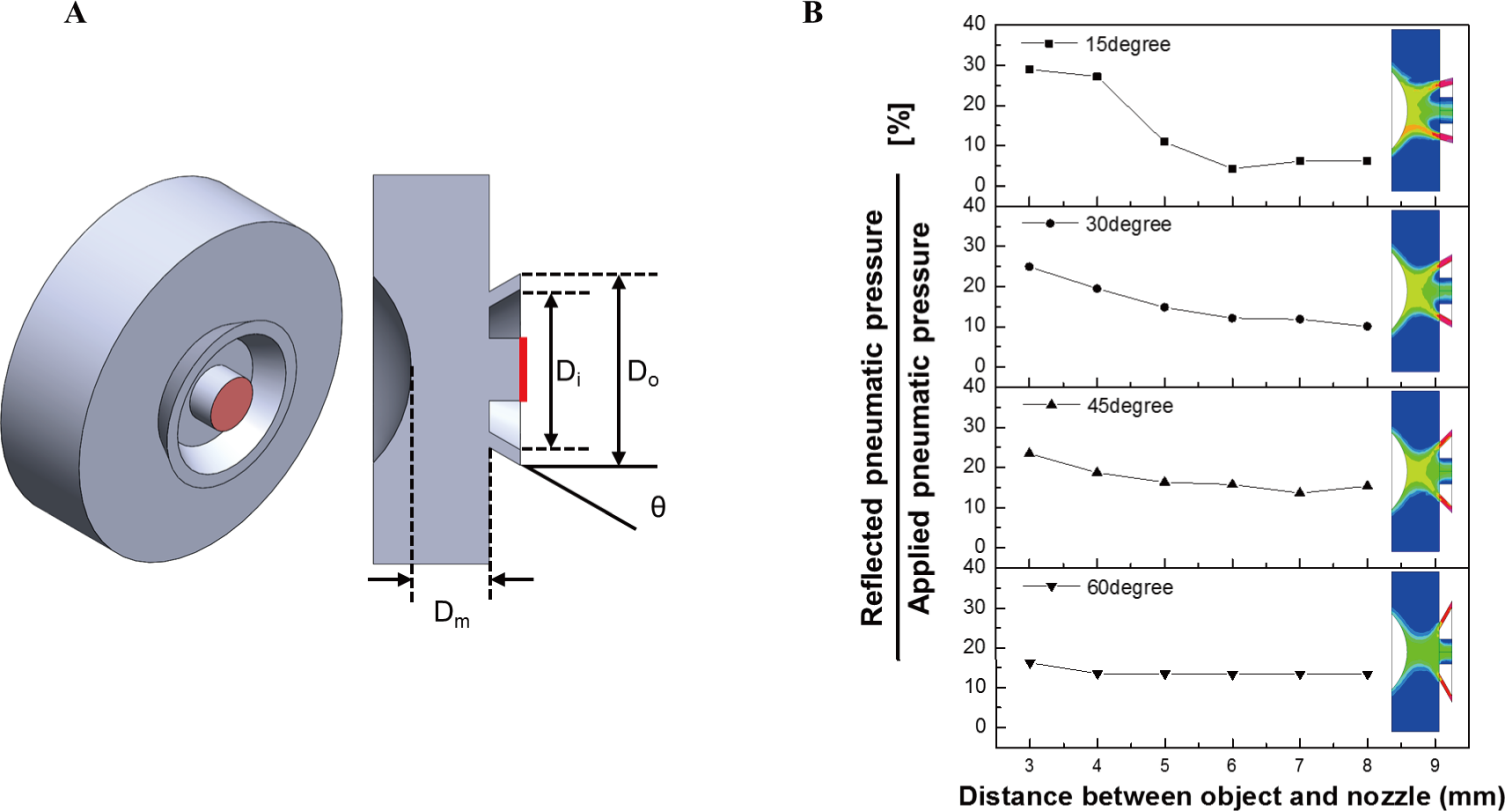
Simulation for prediction of rebounded air pressure by the exit angle of the proposed nozzle. (a) 3-dimensional modeling; (b) Simulation result of rebounded air pressure.

**Fig 4:**
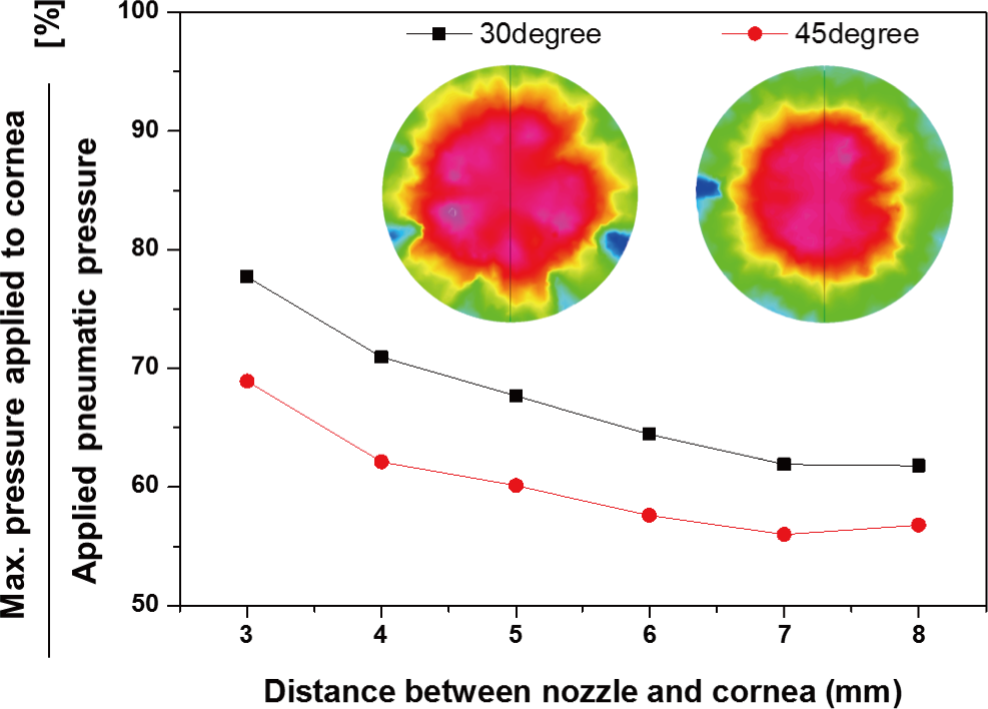
Maximum pressure prediction supplied to cornea by nozzle exit angle of 30 and 45 degree.

An accurate pneumatic pressure is highly important because the non-contact type IOP measuring system proposed in this study uses only the pneumatic pressure to deform the cornea and measure the amount of this deformation. The injected pneumatic pressure should be able to deform the cornea sufficiently after collision with the cornea, and the loss in the reflected pneumatic pressure according to the amount of deformation of the cornea should be minimum. Therefore, a CFD analysis was performed to design an optimal ring-type nozzle and to predict the reflected pneumatic pressure according to the value of θ. Fig 3A shows the three-dimensional flow field and cross-section designed to predict the magnitude of the reflected pneumatic pressure corresponding to the θ of the ring-type nozzle. The internal (D_i_) and external diameter (D_o_) of the ring-type nozzle were designed to be 7 mm and 9 mm, respectively. The distance between the cornea and nozzle, i.e., the measurement distances (D_m_), was between 3–8 mm. As an analysis parameter, θ was changed to 15°, 30°, 45°, and 60°. The cornea was assumed to be rigid, and each analysis was performed under unsteady state conditions using a flow module. As the initial conditions, the velocities along the X, Y, and Z directions were zero, and the atmospheric pressure condition was applied. A pressure of 30 kPa was supplied for 50 ms using a step function. Fig 3B depicts the variation of the reflected pneumatic pressure with respect to the measurement distance based on the θ value. The reflected pneumatic pressure is the pressure at the sensor point located inside the ring-type nozzle. When θ is 15°, a perfect pneumatic pressure curtain is not formed because the pneumatic pressure distribution injected from the nozzle exit was not constant. Therefore, the reflected pneumatic pressure shows a non-uniform decrease rate with the measurement distance. When θ is 60°, the magnitude of the reflected pneumatic pressure is constant according to the distance. It can be confirmed that this is not an output owing to the reflected pneumatic pressure, but a result of the pneumatic pressure injected through the ring-type nozzle being directly introduced into the internal nozzle. On the other hand, the reflected pneumatic pressure of the nozzle with a θ of 30° and 45° was linearly decreased according to distance. As the non-contact IOP measurement system proposed in this study uses the principle of reflection pneumatic distance measurement, the reflected pneumatic pressure should use θ, which decreases linearly with distance, for the sensitivity of the nozzle. The analysis confirmed that a ring-type nozzle with θs of 30° and 45° is advantageous. As shown in Fig 4, the maximum pressure delivered to the cornea at the same injection pneumatic pressure decreases in the form of a second order exponential function depending on the distance. The ring-type nozzle of 30° yields a higher pressure than the ring-type nozzle with θ of 45°. This implies that the cornea can be deformed with a small pressure and therefore, θ was selected as 30°.

### Design of nozzle diameter

The area of the pneumatic pressure injected from the ring-type nozzle is related to the fluid resistance, flow rate, and amount of reflected pneumatic pressure. In order to predict the reflected pneumatic pressure as a function of the area of the injected pneumatic pressure, CFD analysis was performed using the diameter of the nozzle as an analysis parameter.

**Fig 5:**
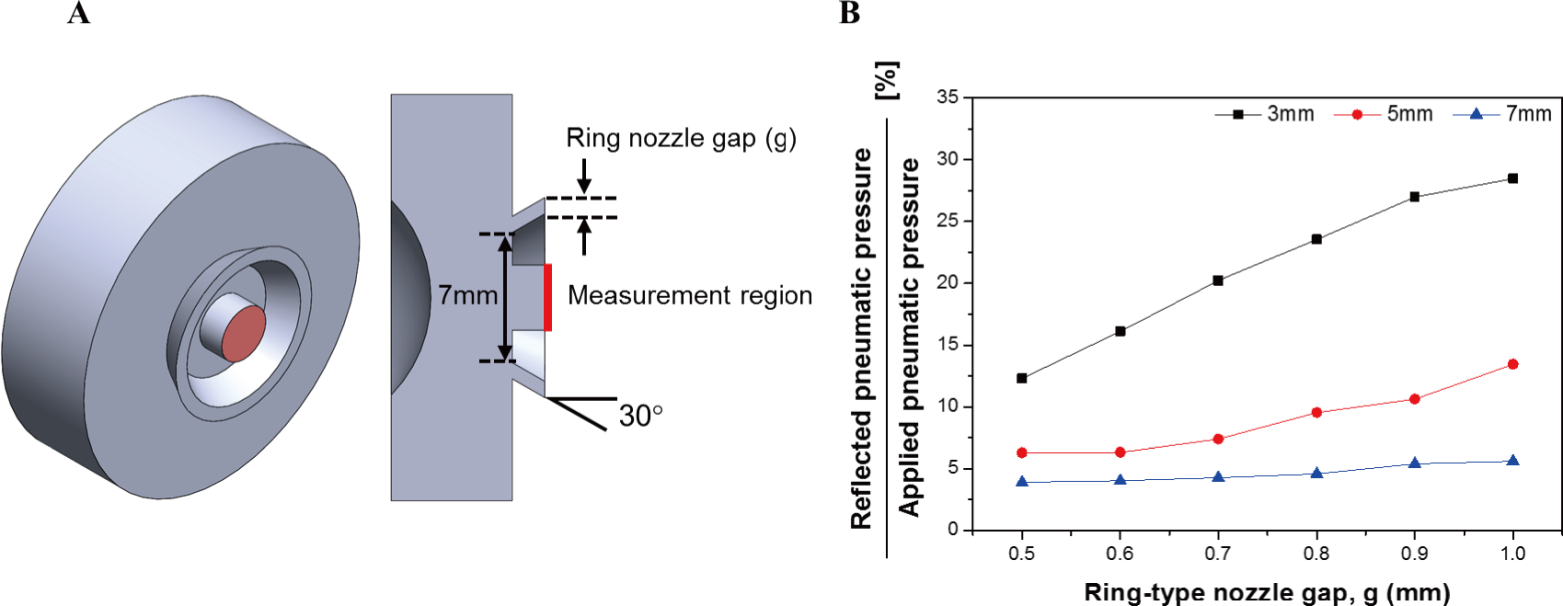
Prediction of reflected pneumatic pressure according to nozzle gap. (a) Flow field to predict the reflected pneumatic pressure by air-puff of 30kPa for step input of 50ms. (b) Graph of the reflected pneumatic pressure using computational fluid dynamics.

Fig 5A shows the flow field used to analyze the reflected pneumatic pressure corresponding to the gap (g) between the internal and external diameters of the ring-type nozzle. The pressure applied from the nozzle was 30 kPa, and θ of the ring-type nozzle was 30°. The distance (i.e., measurement distance) between the cornea and nozzle was 3, 5, and 7 mm, and the reflected pneumatic pressure was confirmed by the average pressure at the position of the sensor inside the ring-type nozzle. The internal diameter of the ring-type nozzle was fixed to 7 mm. The internal diameter is selected in consideration of the size of the pressure sensor to be inserted into the nozzle, and consequently, θ was chosen as 30°. The external diameter of the nozzle was increased from 8 mm to 9 mm at intervals of 0.2 mm. Portable IOP measuring devices are required to be miniaturized, but if the amount of pneumatic pressure used is large, the volume of the pneumatic storage tank increases, so that the distance between the internal and external diameters becomes limited to 1 mm. The plot in Fig 5B shows the results of the analysis according to the gap between the internal and external diameters [25]. As can be seen from the figure, the amount of reflected pneumatic pressure is affected by the injection area of the nozzle. The injection area in turn is related to the flow rate, which is proportional to the reflected pneumatic pressure. However, in this study, the outer diameter of the ring-type nozzle was chosen to be 9 mm considering the volume of the ring-type nozzle and injection amount.

### Fabrication of ring type nozzle

**Fig 6:**
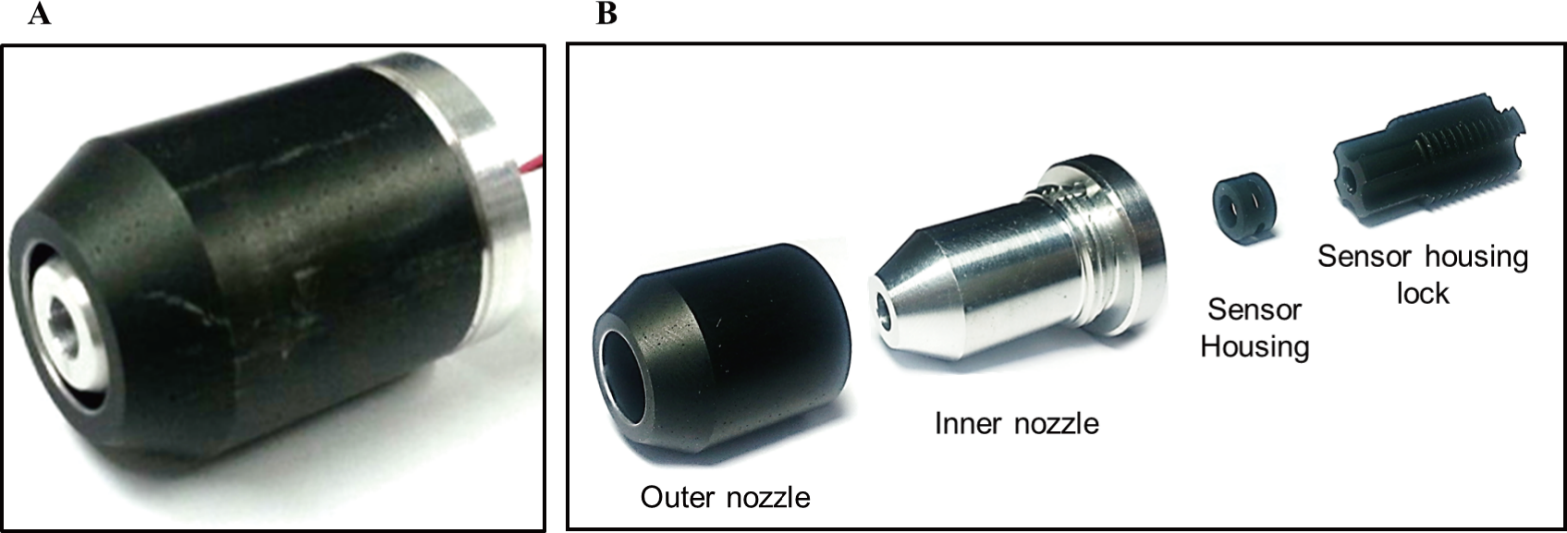
Fabrication of nozzle designed through simulation. (a) Assembly of nozzle. (b) Disassembly of nozzle.

In this study, the ring-type nozzle was designed through CFD analysis. As can be seen in Fig 6A and 6B, the ring-type nozzle is fabricated into four parts by machining of aluminum 6061 [25]. The overall length and diameter are 26 mm and 19 mm, respectively. The outer nozzle is fabricated with a θ of 30° and an internal diameter of 9 mm. Inner nozzle is the part to be inserted in the outer nozzle, and the θ is 30°. The external diameter is fabricated with 7 mm with a nozzle gap of 1 mm. The sensor housing is the part into which the pressure sensor is inserted. As an orifice, four holes are machined for smooth flow into the ring-type nozzle.

### Fabrication of artificial eye

**Fig 7:**
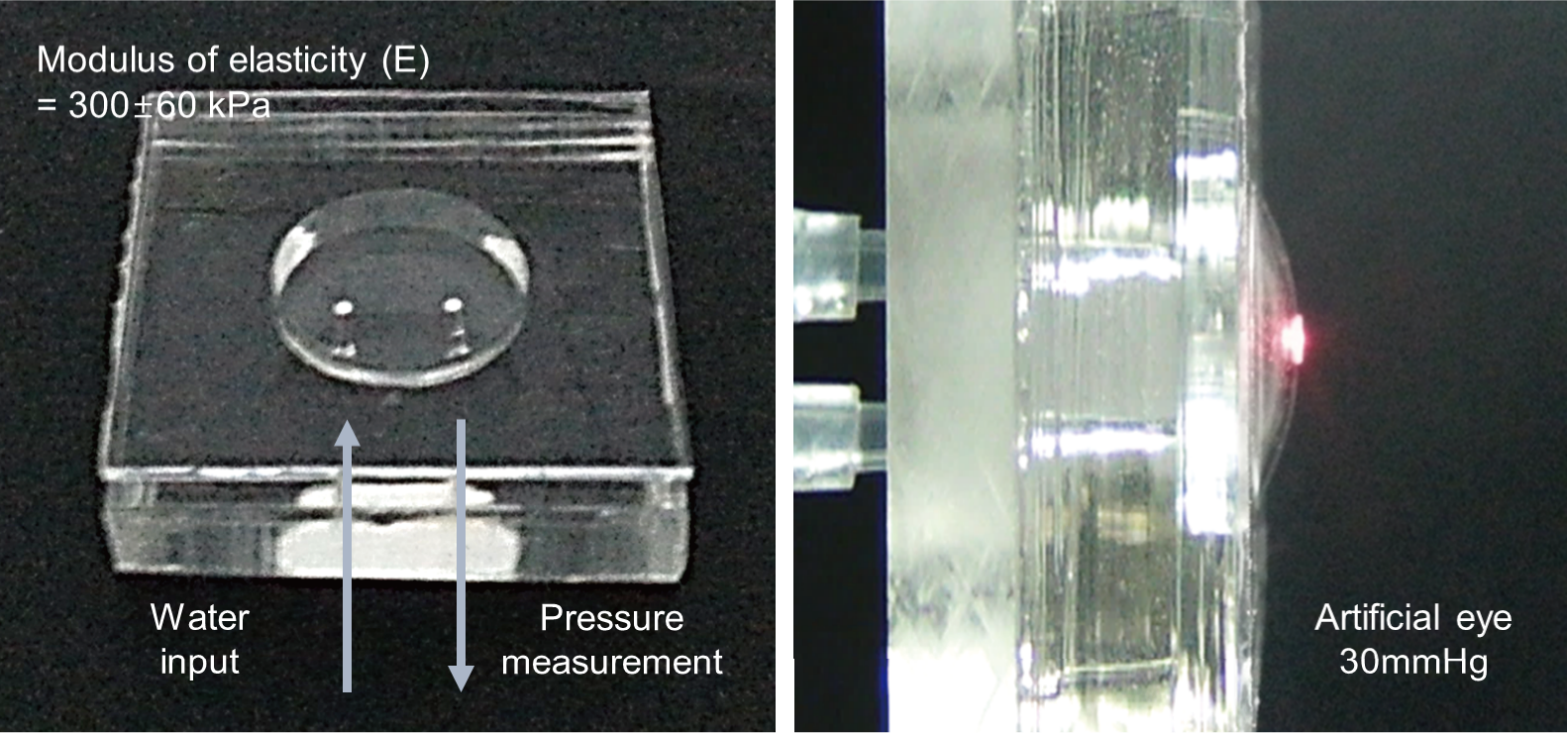
Fabricated artificial eye. Chamber pressure could be controlled by the amount of supplied pressure into the chamber.

**Fig 8:**
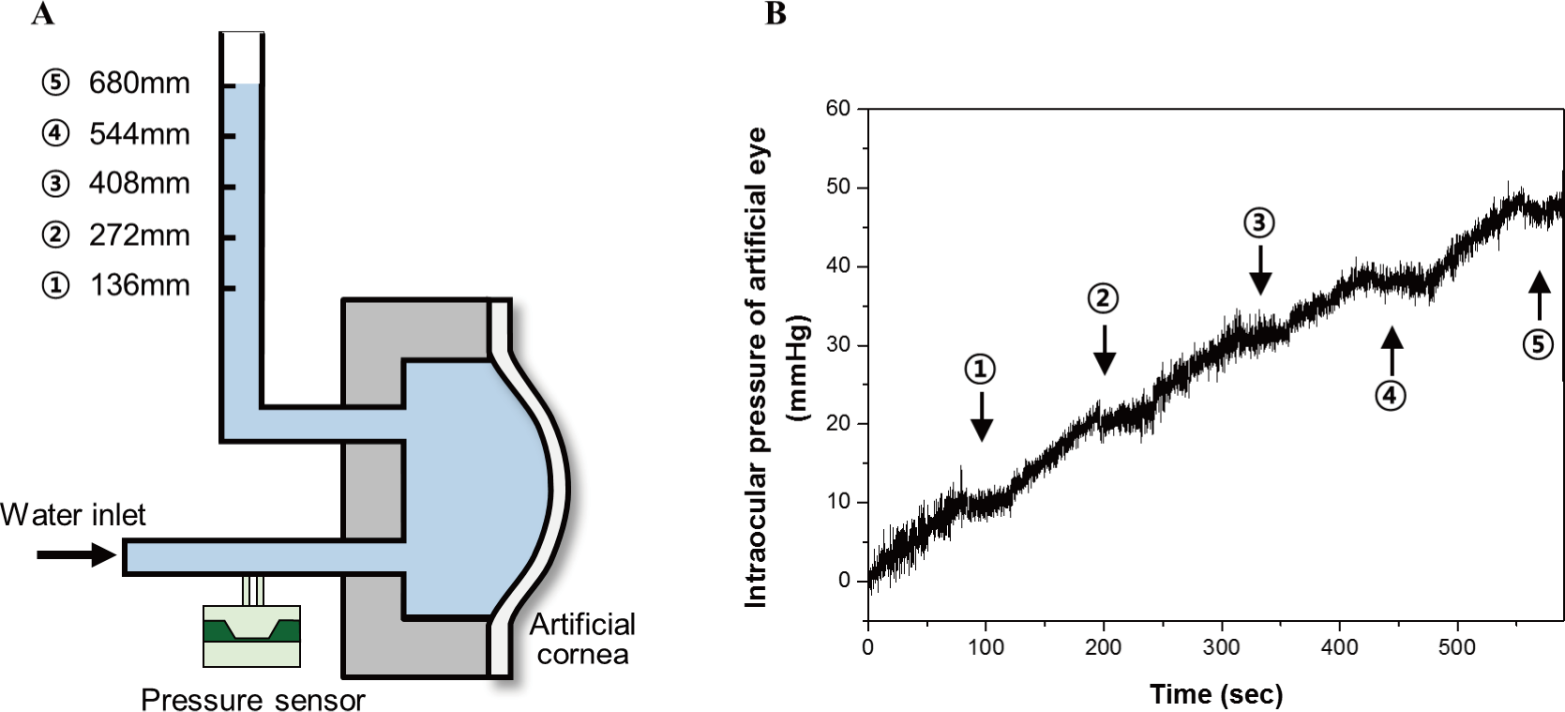
Intraocular pressure adjusting of artificial eye. (a) Schematic view for intraocular pressure supply inside artificial eye used for performance test of nozzle. (b) Intraocular pressure measurement result of artificial eye. Pressure level control using height of water as manometer (136mm = 10mmHg, 272mm = 20mmHg, 408mm = 30mmHg, 544mm = 40mmHg, 680mm = 50mmHg).

As tool to evaluate the performance of the non-contact type IOP system proposed in this study, artificial eyes similar to real eyes were designed and fabricated. The thickness of a human cornea is approximately 0.55 mm, diameter of a cornea is approximately 11 mm, and modulus of elasticity is approximately 290 ± 60 kPa[26]. Fig 7 shows the artificial eyes fabricated using polydimethylsiloxane (PDMS). The corneal thickness of the fabricated artificial eyes is 0.43 mm and it is measured using a laser displacement meter (LC-2440, KEYENCE). The modulus of elasticity is measured to be approximately 300 ± 60 kPa. The elastic modulus of the PDMS membrane used as artificial cornea was measured by the Blister test method using the maximum displacement of the membrane [27]. Deflection under uniform pressure of clamped PDMS membrane is described as

$$P=\frac{16t_{c}^{2}w_{0}}{3(1-\upsilon )r_{ac}^{4}}E+\frac{16\alpha t_{c}w_{0}^{3}}{3(1-\upsilon )r_{ac}^{4}}E$$ (1)

where P, t_c, E, w_0, υ and r_ac are pressure in artificial eye, thickness of artificial cornea, young's modulus of artificial cornea, central deflection of artificial cornea, Poisson’s ratio of artificial cornea and radius of artificial cornea. α is $\frac{\left((1+\upsilon )(173-73\upsilon \right)}{360}$ [28]. In Eq (1), the first term and the second term of the left term are related to small displacement by membrane bending and large displacement by membrane stretching, respectively. Fig 7B displays an artificial eye maintaining an IOP of 30 mm Hg. Water is supplied into the chamber of an artificial eye to play the role of aqueous humor, and the IOP is adjusted using the principle of manometer. Height of water in the tube is adjusted to 136 mm (10 mm Hg), 272 mm (20 mm Hg), 408 mm (30 mm Hg), 544 mm (40 mm Hg) and 680 mm (50 mm Hg), respectively. Fig 8B illustrates a schematic for adjusting the IOP of an artificial eye filled with water (AEW) [25]. Pressure in the artificial eye chamber is measured using a pressure sensor connected to the tube. Fig 8B is a result of measuring the IOP of the artificial eye according to the height of the water in the polymer tube [25]. When the target IOP is 10 mm Hg, 20 mm Hg, 30 mm Hg, 40 mm Hg, and 50 mm Hg, the measured IOP of the artificial eye is 10 mm Hg, 21 mm Hg, 31 mm Hg, 39 mm Hg, and 48 mm Hg, respectively. The performance evaluation of the ring type nozzle was performed by using the AEW adjusted to the respective IOP.

## Results and discussion

**Fig 9:**
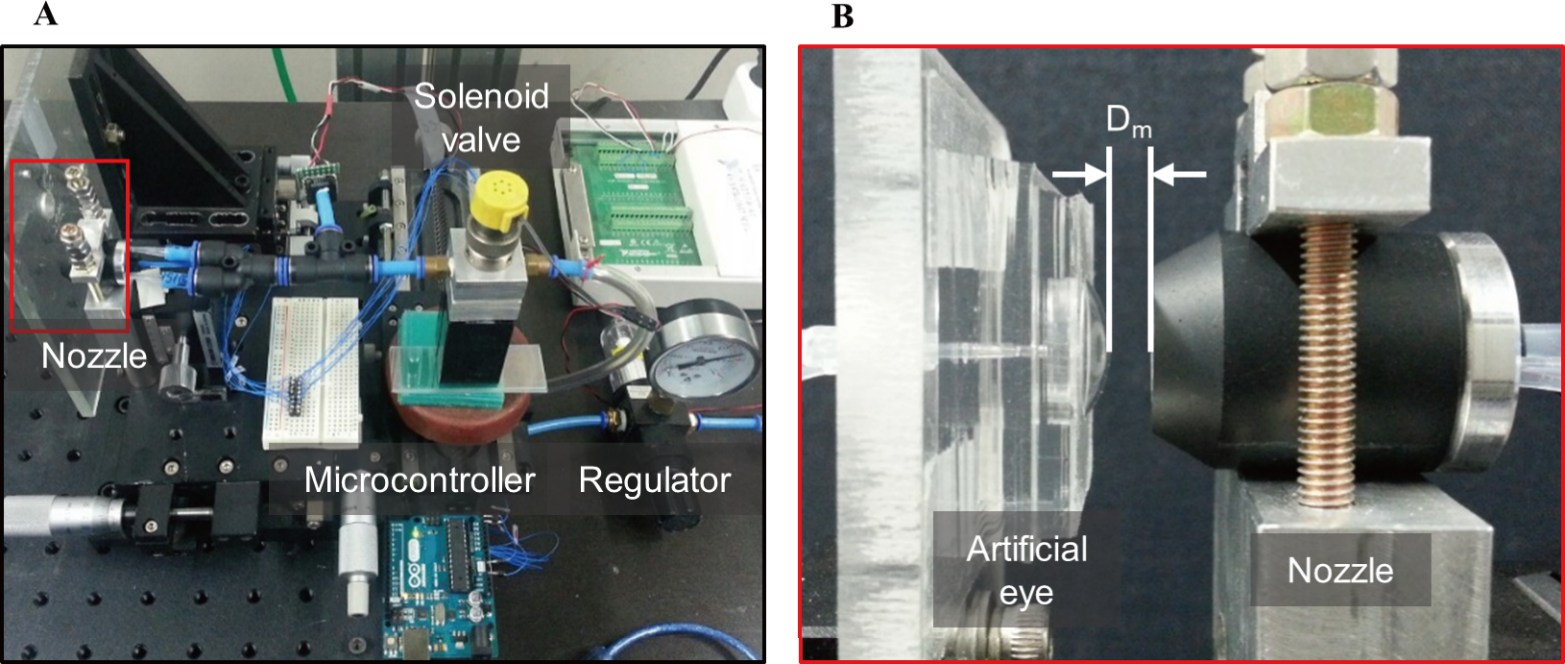
Photograph of experimental apparatus. (a) Overall configuration. (b) Enlarged view of the nozzle and the artificial eye.

Fig 9 shows the experimental apparatus configured for the output comparison corresponding to the distance between the ring-type nozzle with the built-in pressure sensor and a fabricated artificial eye. The experimental apparatus consists of manufactured artificial eyes, ring-type nozzle, solenoid valve, and pneumatic regulator. In a conventional non-contact IOP measurement system, the peak pneumatic pressure applied on the cornea is approximately 25 to 30 kPa (0.25 to 0.30 bar) [29, 30]. The pneumatic pressure is delivered to the solenoid valve at a constant pressure of 30 kPa (0.30 bar) using a regulator, and the solenoid valve injects it into an artificial eye through the ring-type nozzle for 50 ms. Eyelid closing speed is known to be approximately 150 ms. Thus, the pneumatic pressure is injected for 50 ms, i.e., one-third of the speed level [31, 32].

The pressure sensor used for the measurement of the IOP is a digital pressure sensor (MS5803-05BA, TE Connectivity) used as an altimeter. It has a high resolution of 0.036 mbar and can be driven at a low voltage of 3 V. Pressure sensors are used to collect the data in the computer using an inter-integrated circuit (I2C) communication by the Arduino R3. The measurement distance between the nozzle and an artificial eye in the experiment was varied from 3 mm to 5 mm at 0.5 mm intervals. Also, the measuring range of the IOP standard proposed by the International Organization for Standardization (ISO 8612) is 7 to 50 mmHg. Therefore, the IOP of the artificial eyes was adjusted in three steps of 10, 30, and 50 mm Hg.

**Fig 10:**
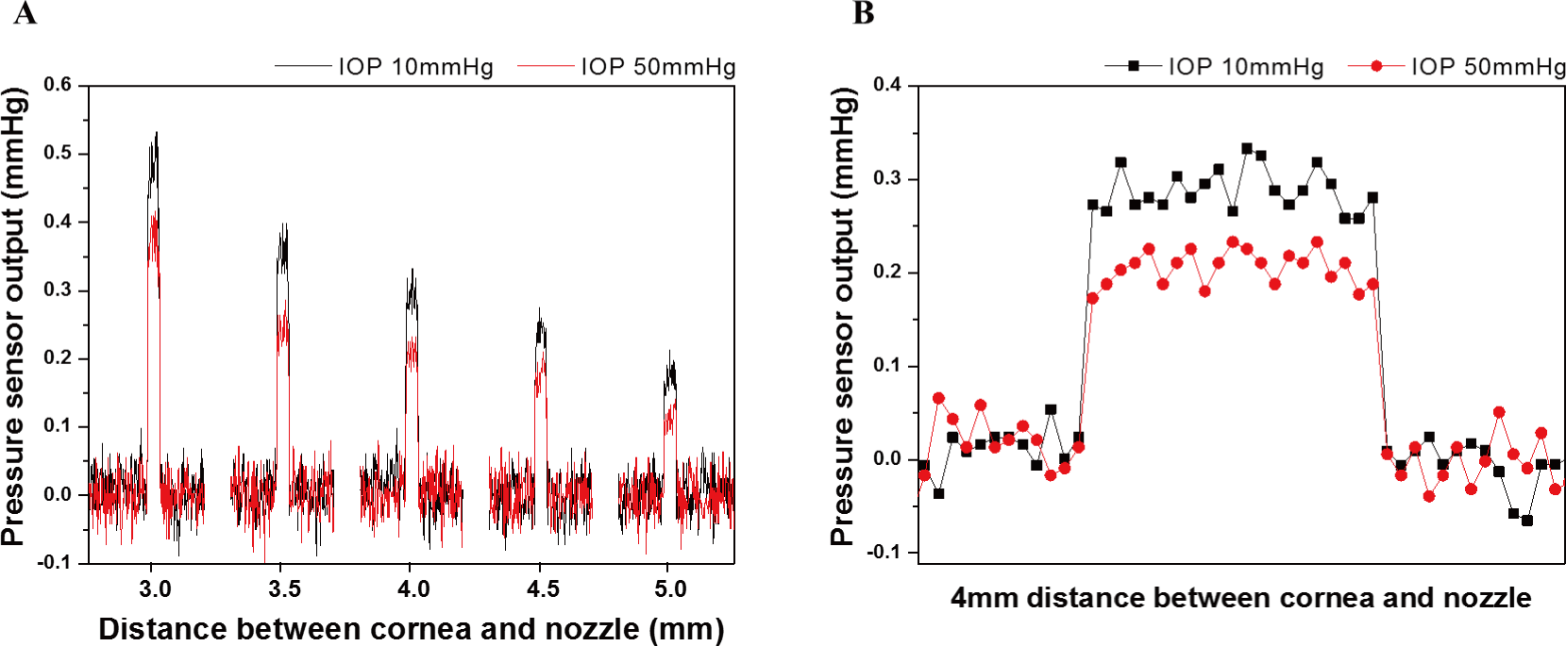
Raw data of pressure sensor about various distance from artificial cornea to nozzle by air-puff. (a) Result from 3.0mm to 5.0mm. (b) Result at 3.0mm distance.

Fig 10A presents the plot comparing the reflected pneumatic pressure about the IOP of 10 mm Hg and 50 mm Hg as the raw data of the pressure sensor measured according to the AEW intraocular pressure and the distance. Fig 10B shows the output of the pressure sensor according to the pneumatic injection time of 50 ms as raw data at 4 mm measurement distance [25]. The reflected pneumatic pressure decreases linearly with increasing distance, and the output is high at a low IOP.

**Fig 11:**
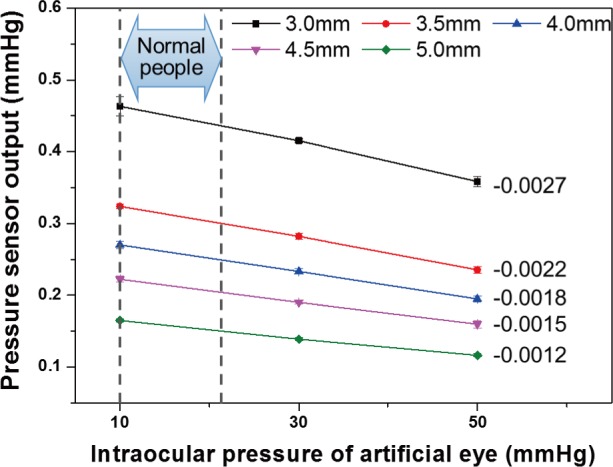
Comparison of reflected pneumatic pressure about the various intraocular pressure and the distance.

Fig 11 displays a graph showing the average of the data measured five times at each distance in order to determine the sensitivity of the ring-type nozzle according to the measurement distance [25]. As shown in the working principle in Fig 2, because a normal person has a lower IOP than the ocular hypertension patient, large corneal deformation occurs. Therefore, the presence or absence of ocular hypertension can be determined by the amount of reflected pneumatic pressure generated according to the difference in the degree of corneal deformation. The experiments show that the reflected pneumatic pressure at a particular distance is inversely proportional to the IOP. (10 mm Hg > 30 mm Hg > 50 mm Hg) This result can be explained by the fact that the corneal deformation is significant at a lower IOP, and there is a greater concentration of the injected pneumatic pressure inside the ring-type nozzle owing to the space created by the deformed cornea. Sensitivity of the ring-type nozzle according to the IOP is -0.0027, -0.0022, -0.0018, -0.0015, and -0.0012 mmHg / mmHg, respectively. The sensitivity (or slope of the graph) displays its decrease with an increase in the measurement distance.

**Fig 12:**
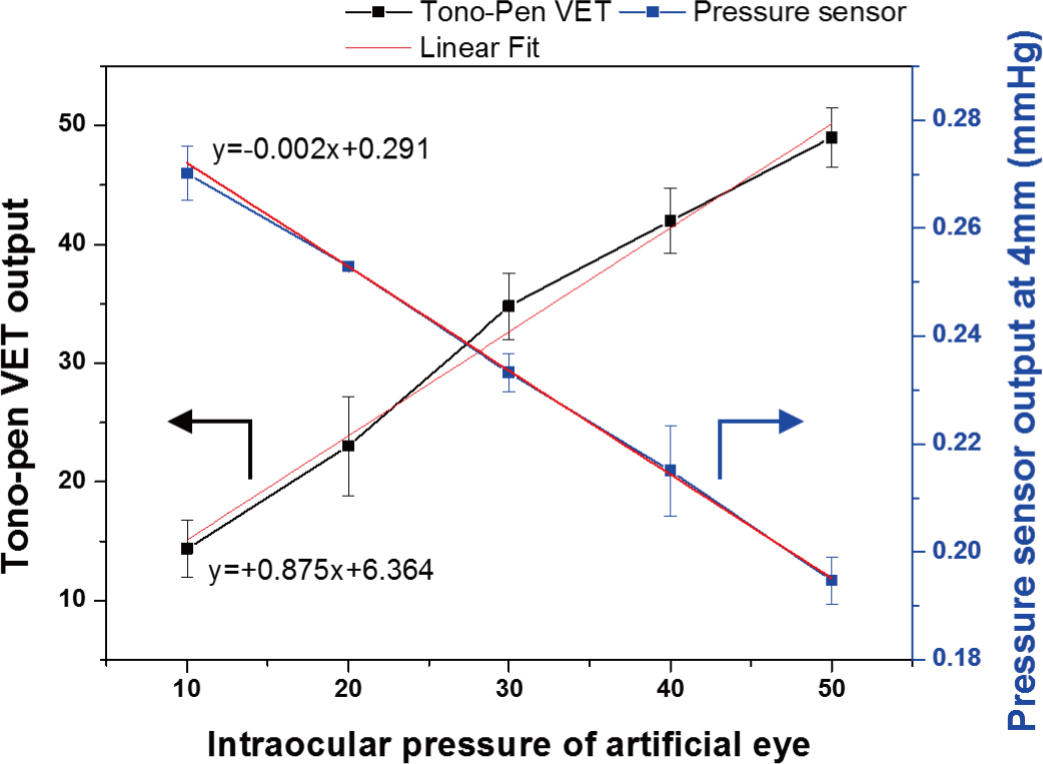
Output comparison of the newly proposed ring nozzle and the commercial tonometer.

Fig 12 is a graph comparing the output of the proposed ring type nozzle with the commercial tonometer (Tono-Pen VET, Reichert, Inc.) using the fabricated artificial eye. The IOP of the artificial eye was adjusted from 10 mmHg to 50 mmHg at 10 mmHg intervals. The Tono-Pen VET are contact type, and use the applanation measurement principle. The result was expressed as average of the data measured five times at each IOP, and the output of Tono-Pen VET increased with increasing IOP of artificial eye. However, The Tono-Pen VET was output about 6.36±1.61 mmHg higher than the IOP of artificial eye. The result of the ring nozzle compared with Tonopen was the result measured at 4mm distance, and the output of the ring nozzle decreased with increasing intraocular pressure in opposition to Tono-Pen VET. Because the cornea was largely deformed at low intraocular pressure, and the pneumatic pressure injected from ring type nozzle was inflow more inside the ring type nozzle by the deformed cornea. Also, the data using the ring type nozzle showed high linearity and the error was smaller than the Tono-Pen VET.

**Fig 13:**
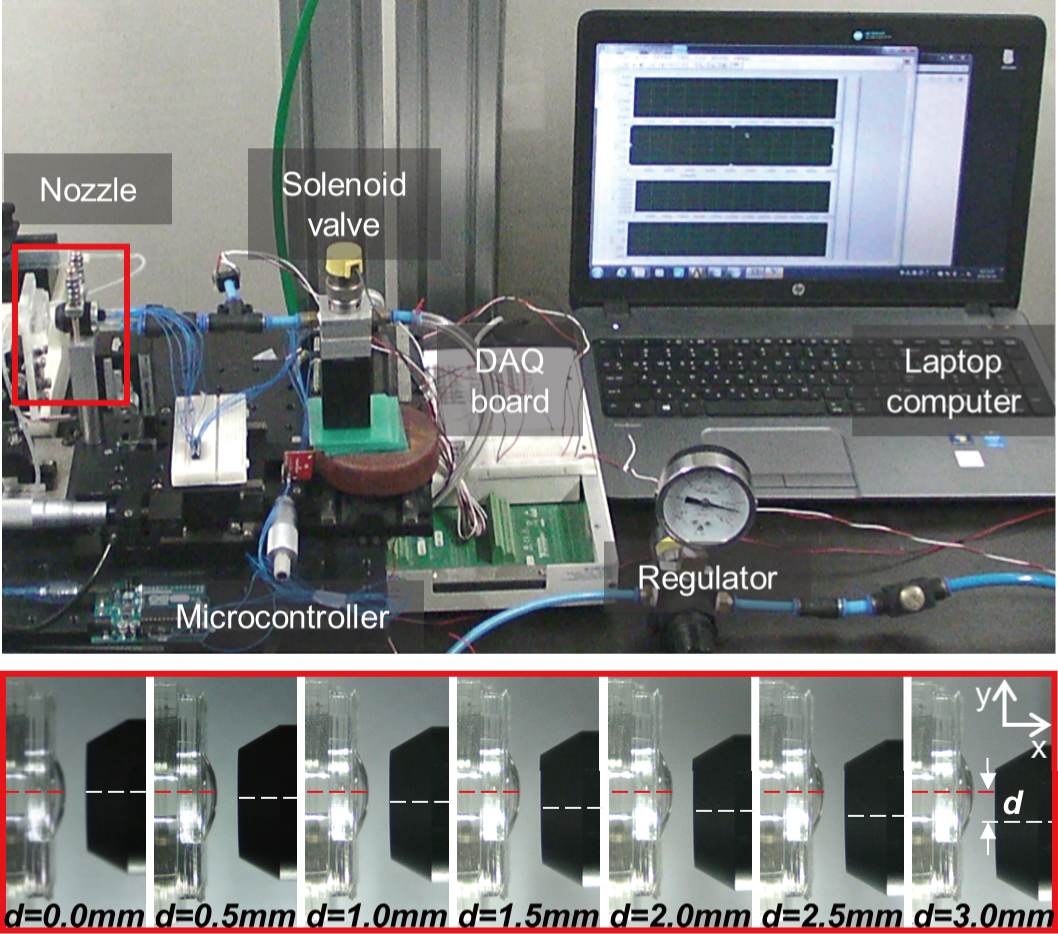
Experiment in misaligned state of artificial eye and nozzle. Overall configuration and enlarged view of misaligned artificial eye and ring nozzle.

**Fig 14:**
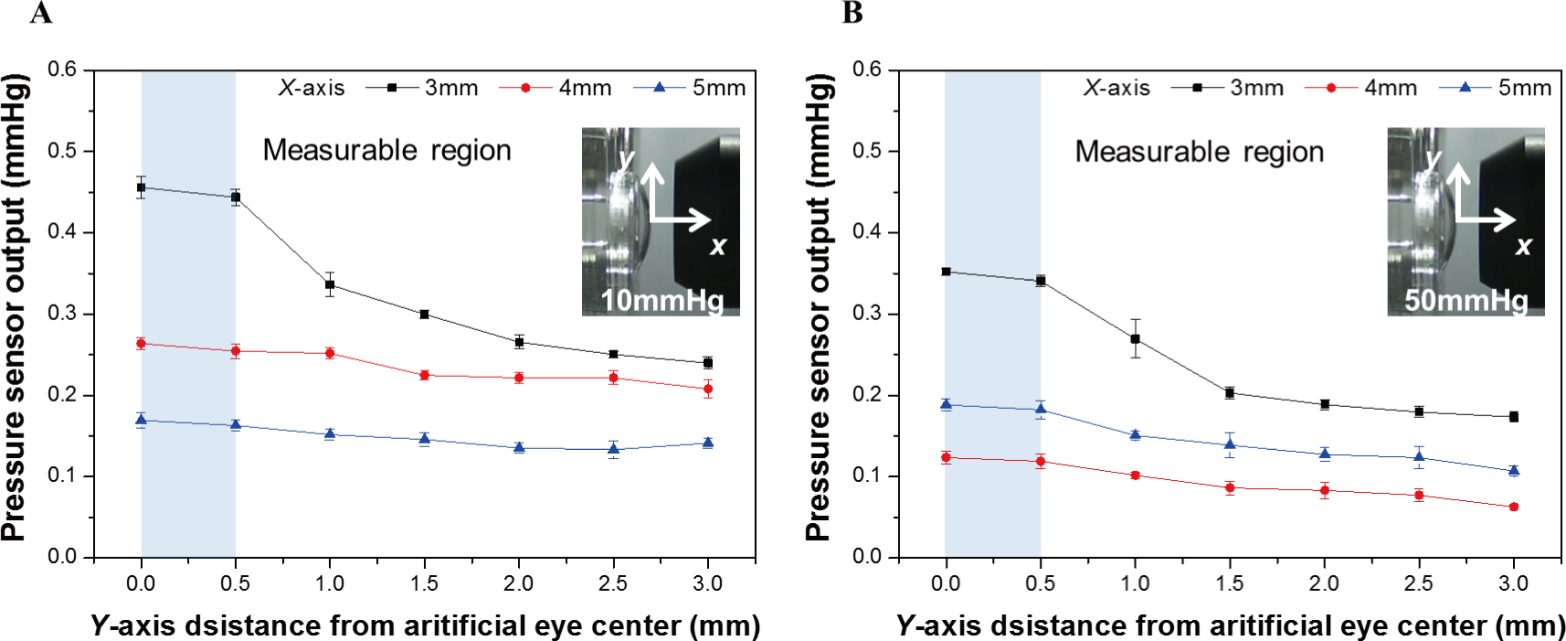
Comparison of reflected pneumatic pressure about misaligned state of artificial eye and nozzle by air-puff. (a) Experiment result about intraocular pressure of 10mmHg. (b) Experiment result about intraocular pressure of 50mmHg.

Fig 13 shows the experimental apparatus constructed to compare the output owing to an alignment error with an artificial eye [25]. The picture below of Fig 13 shows a photograph taken when the misalignment (y-axis) occurs at intervals of 0.5 mm from 0.0 to 3.0 mm (0.0 mm = center alignment) in the y-axis direction at a distance of 3 mm (x-axis) between the artificial eye and ring-type nozzle. The red dotted line is the reference line at the center of the artificial eye, and the white dotted line indicates the reference line at the center of the ring-type nozzle. The IOP of the artificial eye is adjusted to a minimum value of 10 mm Hg and a maximum value of 50 mmHg. The results are expressed as averages of five sets of each experiment. Fig 14A and 14B illustrate the results measured at each alignment error when the IOP of the artificial cornea is 10 mm Hg and 50 mm Hg [25]. The output of the pressure sensor decreases as the measurement distance increases from 3 to 5 mm. When a 0.5 mm alignment error occurred at a measuring distance of 3 mm, the reflected pneumatic pressure at 10 mm Hg and 50 mm Hg based on the center alignment (0 mm) is reduced by 3% and 3.6%, respectively. For alignment errors from 1 mm to 3 mm, the reflected pneumatic pressure is reduced between approximately 26% to 47%. Further, when the measurement distance is 4 mm and 5 mm, the reflected pneumatic pressure linearly increases. From the experimental results, when an alignment error in the range of 1 mm diameter occurs at the measurement distances of 3, 4, and 5 mm, the manufactured ring-type nozzle does not affect the reflected pneumatic pressure. When an alignment error radius was 1 mm (a permissible range 2 mm) at the measurement distances of 3 mm, the accuracy reduction ratio was about 26% or more. However, when an alignment error radius was 1.5 mm (a permissible range 3 mm) at the measurement distances of 4 and 5 mm, the accuracy reduction ratio was about 26% or more, and the acceptable alignment error radius has increased. As a non-contact IOP measuring device, the measuring distance of 4 mm and 5 mm is more advantageous for the user in measuring the intraocular pressure.

## Discussion

In this study, we proposed a non-contact type of IOP measurement system using only the pneumatic pressure to compensate for the disadvantages of a conventional IOP measuring system, and verified the possibility of IOP measurement via a ring type nozzle using fabricated artificial eyes. As an experiment results, the reflected pneumatic pressure was high at low IOP. This result can be explained by the fact that the corneal deformation at a lower IOP was significant, and the injected pneumatic pressure was concentrated more inside the ring type nozzle owing to the space created by the deformed cornea.

The cornea of the artificial eye used for experiment was fabricated with a single thickness for the validation of the newly proposed method. The thickness of the cornea (tc) is a factor affecting the modulus of elasticity. The thicker the thickness, the smaller the displacement of the cornea. Therefore, the amount of deformation of the cornea deformed by the pneumatic pressure becomes small, which will affect the intraocular pressure. In order to analyze the relationship between the cornea thickness and the intraocular pressure, the later experiment should be carried out using thickness of various artificial corneal. In addition, an evaluation of the curvature effect of the cornea should be performed, and finally, an animal experiment for accurate results should be performed.

## Conclusions

Performance evaluation of a fabricated ring type nozzle was conducted based on the measurement distance and IOP using an artificial eye. At 10 mm Hg and 50 mm Hg IOP, the reflected pneumatic pressure at a measurement distance of 5.0 mm decreased by 35% and 33%, respectively compared with the pressure at the measurement distance of 3 mm. The sensitivity of the ring type nozzle according to the intraocular pressure at the measurement distance from 3.0 to 5.0 mm was -0.0027, -0.0022, -0.0018, -0.0015, and -0.0012 mm Hg/mm Hg, respectively. We confirmed via experiments that the IOP values of normal person and ocular hypertension patients can be distinguished. Furthermore, when a misalignment occurred between the artificial eye and ring nozzle, the reflected pneumatic pressure did not affect the reflected pneumatic pressure within an alignment error range of ± 0.5 mm from the center of an artificial eye. In this study, we proposed a portable non-contact type of IOP measurement system using only the pneumatic pressure to compensate for the disadvantages of a conventional IOP measuring system, and verified the possibility of IOP measurement via a ring type nozzle using fabricated artificial eyes. Also, IOP measurement method proposed in the study is unlike conventional non-contact type tonometer, expensive optical modules are not needed because the intraocular pressure is measured using only pneumatic pressure, and the volume of instrument can also be reduced. The pressure sensor uses a commercialized product, and the ring-type nozzle, which is a core part, has advantageous that it can be manufactured at low cost through simple mechanical machining or injection molding.

However, this system needs to know the distance between the cornea and the nozzle to predict the accurate intraocular pressure. In order to avoid a mistaken diagnosis, we can consider two different pneumatic pressure injections as the first pneumatic pressure for distance measurement and the second pneumatic pressure for IOP prediction.
